# Sparse Sliding-Window Kernel Recursive Least-Squares Channel Prediction for Fast Time-Varying MIMO Systems

**DOI:** 10.3390/s22166248

**Published:** 2022-08-19

**Authors:** Xingxing Ai, Jiayi Zhao, Hongtao Zhang, Yong Sun

**Affiliations:** 1 ZTE Corporation, Algorithm Department, Wireless Product R&D Institute, Wireless Product Operation Division, Shenzhen 518057, China; 2 Key Laboratory of Universal Wireless Communications, Ministry of Education of China, Beijing University of Posts and Telecommunications, Beijing 100876, China

**Keywords:** channel prediction, time-varying channels, MIMO system, kernel methods, recursive least squares

## Abstract

Accurate channel state information (CSI) is important for MIMO systems, especially in a high-speed scenario, fast time-varying CSI tends to be out of date, and a change in CSI shows complex nonlinearities. The kernel recursive least-squares (KRLS) algorithm, which offers an attractive framework to deal with nonlinear problems, can be used in predicting nonlinear time-varying CSI. However, the network structure of the traditional KRLS algorithm grows as the training sample size increases, resulting in insufficient storage space and increasing computation when dealing with incoming data, which limits the online prediction of the KRLS algorithm. This paper proposed a new sparse sliding-window KRLS (SSW-KRLS) algorithm where a candidate discard set is selected through correlation analysis between the mapping vectors in the kernel Hilbert spaces of the new input sample and the existing samples in the kernel dictionary; then, the discarded sample is determined in combination with its corresponding output to achieve dynamic sample updates. Specifically, the proposed SSW-KRLS algorithm maintains the size of the kernel dictionary within the sample budget requires a fixed amount of memory and computation per time step, incorporates regularization, and achieves online prediction. Moreover, in order to sufficiently track the strongly changeable dynamic characteristics, a forgetting factor is considered in the proposed algorithm. Numerical simulations demonstrate that, under a realistic channel model of 3GPP in a rich scattering environment, our proposed algorithm achieved superior performance in terms of both predictive accuracy and kernel dictionary size than that of the ALD-KRLS algorithm. Our proposed SSW-KRLS algorithm with M=90 achieved 2 dB NMSE less than that of the ALD-KRLS algorithm with v=0.001, while the kernel dictionary was about 17% smaller when the speed of the mobile user was 120 km/h.

## 1. Introduction

Multiantenna technology can fully use spatial dimension resources and dramatically improve the capacity of wireless communication systems without increasing transmission power and bandwidth [1]. Meanwhile, beamforming technology [2,3] is widely used to reduce the interference between cochannel users with cooperative transmission and reception since it can compensate channel fading and distortion caused by the multipath effect. Base stations optimize the allocation of radio resources through reasonable precoding, rendering the desired signal and interference more orthogonal provided that CSI is known at the base station. Thus, the acquisition of CSI is very important in the cooperation of transmission and reception [4]. However, due to the dynamics of the channel, especially when the terminal is moving at a high speed, the acquisition of CSI is a formidable problem in MIMO systems.

In TDD systems, the user sends a sounding reference signal (SRS), and the base station performs channel estimation algorithms such as LS [5] and MMSE [6]. Then, the obtained CSI is used for downlink beamforming to realize cooperative signal processing. The coherence time of wireless channels is the time duration after which CSI is considered to be outdated. When the terminal is moving at a high speed, the Doppler frequency shift grows, and the time variability of the channel is severe, which leads to the shortening of channel coherence time. The measured uplink channel CSI cannot represent the real channel state of downlink slots, resulting in the mismatch between the downlink beamforming designed according to the measured CSI and the actual channel. In [1], with a typical CSI delay of 4 milliseconds, the user terminal speed at 30 km/h led to as much as 50% performance reduction versus in the low-mobility scenario at 3 km/h.

In order to overcome the performance degradation caused by severe time-varying channels, [7] proposed a tractable user-centric CSI model and a robust beamforming design by taking deterministic equivalents [8] into account. However, when the user terminal is at high speed, the channel shows nonstationary characteristics, and the statistical characteristics of the channel also change with time, which cannot be used for the beamforming of high-speed time-varying channels.

Another approach is to use a channel prediction algorithm [9,10,11,12,13,14,15,16] to obtain more accurate CSI for beamforming. The kernel method is widely used in channel prediction algorithms due to its ability to track nonlinear channels, and its adaptation to time-varying channels [17,18,19,20,21,22]. However, algorithms based on kernel methods face the problem in online prediction that the networks structure grows as the size of the training samples grows in time. Though researchers have proposed sparseness methods to limit the size of samples by setting prerequisites for new samples added into the dictionary, the size of the kernel dictionary cannot be precisely controlled. Therefore, this paper proposes a new channel algorithm based on the kernel method that maintains the size of the kernel dictionary within a fixed budget while precisely tracking the fast time-varying dynamic characteristics.

### 1.1. Related Work

State-of-the-art channel fading prediction algorithms were reviewed in [9]. Parametric radio channel-based methods [10] consider channel changes faster than multipath parameters, and the estimation of these parameters can help in the extrapolation of the channel into future. However, the effective time of static multipath parameters is inversely proportional to the terminal moving speed, rendering the channel prediction based on radio parameters not appropriate for high-speed scenarios. Autoregressive (AR) model-based methods [11,13] do not explicitly model physical scatterings, considering the time-varying channel as a stochastic wide-sense stationary (WSS) process and the temporal autocorrelation function is used for prediction. Nevertheless, they are not capable of predicting ephemeral variations in non-wide-sense-stationary (NWSS) channels due to their linear correlation assumption. When treating NWSS channels, many studies attempted to use machine learning in channel prediction. The authors in [14] proposed a backpropagation (BP) framework regarding channel prediction for backscatter communication networks that considers both spatial and frequency diversity. In [15], the authors developed a machine-learning method for predicting a mobile communication channel on the basis of a specific type of convolutional neural network. Although these algorithms can achieve good performance, they need to build neural networks, require a large number of samples for training, and the complexities of these algorithms are high. The channel prediction method based on a support vector machine [16,23] uses Mercer’s theorem [24] to map the channel sample space to the high-dimensional space, and performs linear regression in the high-dimensional space to solve the problem of tracking nonlinear channels well. The kernel recursive least-squares (KRLS) [17,18,19,20,21,22] algorithm is a nonlinear version of the recursive least-squares (RLS) algorithm. It can not only solve nonlinear problems, but also adaptively iterate the model parameters.

In order to solve this problem and render the online kernel method feasible, researchers proposed various sparseness methods or criteria, such as approximate linear dependency (ALD) [17], the novelty criterion (NC) [20], the surprise criterion (SC) [21], and the coherence criterion (CC) [22]. On the basis of these sparseness methods or criteria, only new input samples that meet the prerequisites are added to the dictionary. However, these sparse methods cannot precisely control the size of the kernel dictionary, which motivated us to introduce a sliding window to control the size of the kernel dictionary. Recently, some KRLS-based algorithms with an inserted forgetting factor have achieved better performance than that of QKRLS algorithms [25,26] and ALD-KRLS, which motivated us to insert a forgetting factor into the proposed SSW-KRLS algorithm. This paper proposes a new sparse sliding-window KRLS algorithm where a candidate discard set is selected through correlation analysis between the mapping vectors in the kernel Hilbert spaces of the new input sample and the existing samples in the kernel dictionary; then, the discarded sample is determined in combination with its corresponding output to achieve dynamic sample updates.

### 1.2. Contributions

The main contributions of this paper are summarized as follows:

- We propose a novel sparse sliding-window KRLS algorithm. To precisely control the size of samples, we introduce a sample budget as a size restriction. When the dictionary was smaller than the sample budget, we directly added the new sample to the dictionary. Otherwise, we chose the best sample to discard according to our proposed new criterion.
- To differentiate the sample value collected at different times, we introduced a forgetting matrix. By setting different forgetting values for samples collected at different times, we quantified the time value of the samples. The older sample had a smaller forgetting value, which means that its time value was smaller. In this way, we considered both the correlation of samples and the time value when discarding old samples.
- Regarding our new method for discarding old samples, we set a candidate set where we decided which sample to discard. The candidate set was obtained by adding the samples that had larger kernel functions of the new sample than a threshold and were highly correlated with the new sample. Then, we conducted a weighted estimation of the output value of these samples. We decided which sample to discard on the basis of the deviation between its output and the estimated value.

## 2. System Model

**Figure 1:**
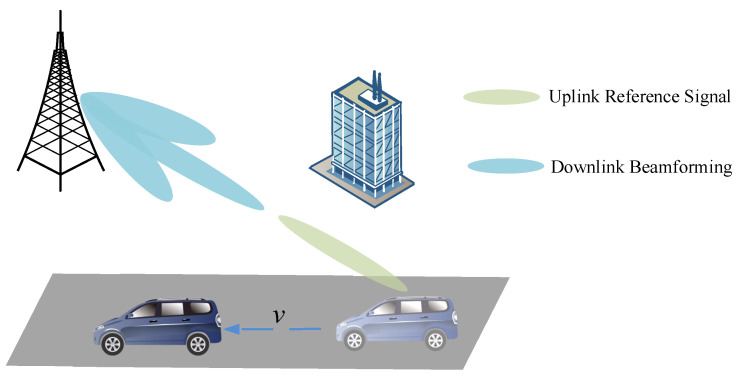
In TDD mobile communication systems, the beamforming performance of high-speed mobile terminal worsens. The user sends a SRS, and the base station runs channel estimation algorithms and performs beamforming. However, due to the user’s movement, the terminal can only obtain sidelobe gain in the downlink slots.

We considered two typical user movement scenarios: urban road where the moving speed of users is 60 km/h, and a highway where the moving speed of users is 120 km/h. As shown in Figure 1, in TDD systems, the user sends a SRS, and the base station runs channel estimation algorithms such as LS and MMSE. The channel matrix is first estimated in frequency-domain; then, the channel frequency response is transformed into the time-domain by IDFT. The noise is evenly distributed in the whole time domain and is easy to be eliminated using a raised cosine window. Therefore, the effect of noise is very small compared to the user’s high mobility. Due to the channel reciprocity in TDD systems, uplink channel CSI can be directly used in the design of downlink beamforming to realize cooperative signal processing. However, nonstationary fast fading characteristics of mobile environments bring challenges to multi-input multi-output (MIMO) communications.

**Figure 2:**
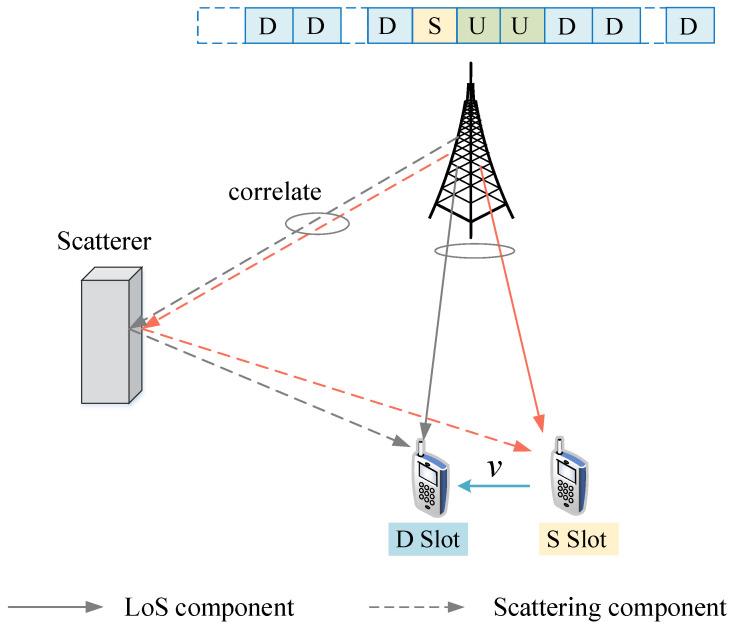
In TDD mobile communication systems, the interval between slots S and D is short in two adjacent SRS periods, and the LoS and scattering components are correlated.

When the terminal is moving at a high speed, the Doppler frequency shift grows, and the time variability of the channel is severe. The measured uplink channel CSI cannot represent the real channel state of the downlink slots, resulting in performance degradation, and a mismatch between the measured CSI and the actual channel. As shown in Figure 2, during the SRS reporting cycle, the user can be regarded as moving in a fixed direction at a fixed speed. In two adjacent time slots, due to the short moving distance of the user, the amplitude and phase of the direct and scattered components have a certain correlation. When the user speed is low, an AR-based channel prediction method can achieve good performance by utilizing the linear correlation between adjacent time slots. However, when the user is moving at a high speed, the channel correlation between adjacent time slots presents nonlinear characteristics. Kernel methods are needed to exploit the nonlinear correlation characteristics of the channel.

**Figure 3:**
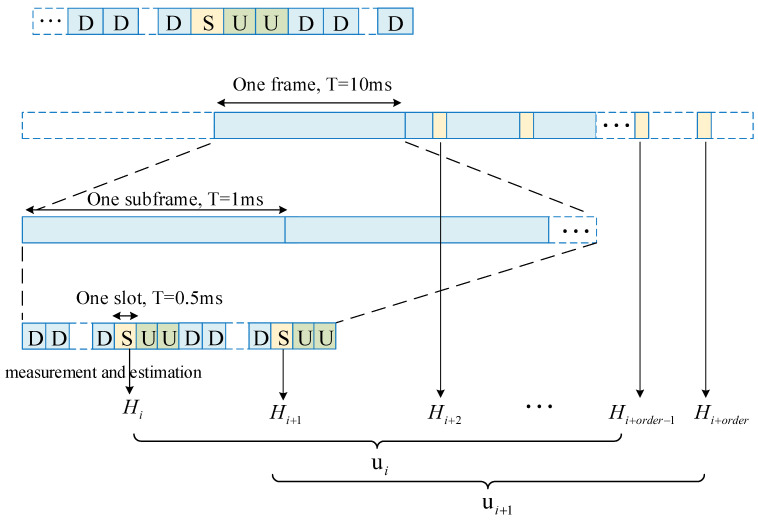
An illustration of the input and output pairs in a channel prediction module.

The user sends SRS in special time slots, and the BS performs the channel measurement and estimation to obtain the channel matrix. As shown in Figure 3, the channel matrix measured by BS in the *i*-th SRS period is assumed to be $H_{i}\in C^{N_{r}\times N_{t}}$. $N_{t}$ and $N_{r}$ represent the number of antennas of the BS and the mobile UE, respectively. The real and imaginary parts are separately processed in the subsequent algorithm, and *H* represents a real matrix in the later representation. The prediction order means that each channel matrix is related to the channel matrix measured in the previous-order SRS periods, so the input vector of the prediction system is

(1) $$u_{i}=\left[H_{i},H_{i+1},\ldots ,H_{i+order-1}\right].$$

There are some complex and unknown dependencies between $u_{i}$ and $H_{i}$: $H_{i+order-1}=$$f\left(u_{i}\right)=f\left(\left[H_{i},H_{i+1},\ldots ,H_{i+order-1}\right]\right)$ that need to be exploited by kernel methods.

## 3. Traditional KRLS Algorithm

In this section, we introduce the traditional KRLS algorithm and several extensions to the KRLS algorithm.

### 3.1. Traditional KRLS Algorithm

Assume a set of ordered input-output pairs $D=\left\{u_{i},y_{i}\right\}{|}_{i=1}^{m}$, where *m* is the total number of samples, $u_{i}\in R^{m}$ are m-dimensional input vectors and $y_{i}\in R$ is the output. We call *D* a dictionary that records the input-output pairs collected before the *i*-th time slot. First, according to the Mercer theorem, we can adopt a nonlinear function φ(·) to transform data $u_{i}\in R^{m}$ into a high-dimensional feature space: $\varphi (\cdot ):R^{m}\to R^{c}$. The corresponding kernel function is $\kappa \left(u,v\right)=\langle \varphi \left(u\right),\varphi \left(v\right)\rangle$. We need to minimize the cost function:

(2) $$J=\sum_{j=1}^{i}{\left|y_{i}-w_{i}\varphi_{j}\right|}^{2}={∥\Phi {{}_{i}}^{T}w_{i}-y_{i}∥}^{2},$$

where $\Phi_{i}=\left[\varphi \left(u_{1}\right),...,\varphi \left(u_{i}\right)\right]=\left[\varphi_{1},\ldots ,\varphi_{i}\right]$ is a high-dimensional matrix. By minimizing the cost function as (2), we can obtain weight $w_{i}$ = $\Phi {}_{i}^{†}y_{i}$, where $\Phi_{i}^{†}$ is the pseudoinverse of $\Phi_{i}$. $\Phi {}_{i}^{†}$ cannot be solved directly because kernel function $\kappa \left(u,v\right)$ is a high-dimensional mapping, and the exact mapping function is unknown. To avoid overfitting when the samples are small, we used L2 regularization, so the cost function is reformulated as:

(3) $$J={∥\Phi {{}_{i}}^{T}w_{i}-y_{i}∥}^{2}+\lambda {∥w_{i}∥}^{2}.$$

By letting $\frac{\partial J}{\partial w_{i}}=0$, we can obtain that

(4) $$w_{i}=\Phi {}_{i}{\left[\lambda I+\Phi {}_{i}^{T}\Phi {}_{i}\right]}^{-1}y_{i}=\Phi {}_{i}\alpha \left(i\right).\;$$

By substituting (4) into (3), the cost function can be reformulated as:

(5) $$J={∥\Phi {{}_{i}}^{T}\Phi {}_{i}\alpha \left(i\right)-y_{i}∥}^{2}+\lambda {∥w_{i}∥}^{2}={∥K_{i}\alpha \left(i\right)-y_{i}∥}^{2}+\lambda {∥w_{i}∥}^{2},$$

where $K_{i}$ is the kernel matrix and the element located at the *i*-th row and *j*-th column of $K_{i}$ is $K_{i}\left(i,j\right)=\kappa \left(u_{i},u_{j}\right)$. The problem is to solve

(6) $$\alpha \left(i\right)={\left[\lambda I+\Phi {}_{i}^{T}\Phi {}_{i}\right]}^{-1}y_{i}={\left[\lambda I+K_{i}\right]}^{-1}y_{i}=Q\left(i\right)y_{i},$$

Then, the inverse of Q(i) can be obtained as:

(7) $$Q{\left(i\right)}^{-1}=\lambda I+K_{i}=\left[\begin{array}{cc} Q{\left(i-1\right)}^{-1} & k_{i}\\ {k_{i}}^{T} & \lambda +\kappa \left(u_{i},u_{i}\right) \end{array}\right],$$

where $k_{i}={\Phi_{i-1}}^{T}\varphi_{i}$. Thus, Q(i) can be solved using the inversion of the partitioned matrix.

(8) $$Q\left(i\right)=r{\left(i\right)}^{-1}\left[\begin{array}{cc} Q\left(i-1\right)r\left(i\right)+z\left(i\right)z{\left(i\right)}^{T} & -z(i)\\ -z{\left(i\right)}^{T} & 1 \end{array}\right],$$

where $z\left(i\right)=Q\left(i-1\right)k_{i}$ and $r\left(i\right)=\lambda +{\varphi_{i}}^{T}\varphi_{i}-z{\left(i\right)}^{-1}\varphi_{i}$.

The traditional KRLS algorithm is summarized in Algorithm 1.
**Algorithm 1** Traditional KRLS algorithm.1:Initialize $Q\left(1\right)={\left(\lambda +k(u_{1},u_{1})\right)}^{-1}$ and $\alpha \left(1\right)=Q\left(1\right)y_{1}$.2:Iterate for i > 1: $k_{i}={\Phi_{i-1}}^{T}\varphi_{i}$, $z\left(i\right)=Q\left(i-1\right)k_{i}$, $r\left(i\right)=\lambda +{\varphi_{i}}^{T}\varphi_{i}-z{\left(i\right)}^{-1}\varphi_{i}$,
$$Q\left(i\right)=r{\left(i\right)}^{-1}\left[\begin{array}{c} Q\left(i-1\right)r\left(i\right)+z\left(i\right)z{\left(i\right)}^{T}-z(i)\\ -z{\left(i\right)}^{T}\;\;\;1\;\; \end{array}\right]$$

By relying on the kernel trick, the traditional KRLS algorithm can deal with nonlinear problems by nonlinearly transforming data into a high-dimensional reproducing kernel Hilbert space, which is similar to other techniques such as support vector machines (SVMs). Compared to SVMs, it avoids large-scale high-dimensional computing through iterative computations. However, the traditional KRLS algorithm grows linearly with the number of processed data, rendering growing complexities for each consecutive update if no additional measures are taken. In order to render online kernel algorithms feasible, growth is typically slowed down by approximately representing the solution using only a subset of bases that are considered to be relevant according to a chosen criterion. In our proposed SSW-KRLS algorithm, we took similar operations to avoid an increase in computation, which is discussed in detail in Section 4.

### 3.2. Extensions to KRLS Algorithm

The kernel recursive least-squares method is one of the most efficient online kernel methods, and it achieves good performance in nonlinear fitting and prediction. However, the bottleneck problem is that the network structure grows with the training samples, which leads to insufficient memory and computational complexities when processing continuously incoming signals. In order to solve this problem and render the online kernel method feasible, researchers have proposed various sparseness methods or criteria, such as approximate linear dependency (ALD), the novelty criterion (NC), the surprise criterion (SC) and the coherence criterion (CC). On the basis of these sparseness methods or criteria, only new input samples that meet the prerequisites are used as training samples. Thus, the growth of network structure is effectively slowed down. Table 1 shows several common sparseness criteria.

The performance of the above methods in filtering samples largely depends on the selected threshold. As time goes by, the number of samples increases slowly and lastly stabilizes, while the update of model parameters tends to be slow. This is not conducive to updating time-varying channels. Furthermore, when the user moves into a new environment, the outdated samples are reserved, which is not conducive to channel prediction.

An effective way to keep the dictionary updated while precisely controlling its size is using the sliding window. A simple implementation is discarding the oldest sample directly each time a new sample is collected, but it lacks the judgement of correlation between samples. Sparseness simply based on time information is unreliable and may result in unstable prediction performance when the window is small. In order to solve this problem, we propose a new algorithm, SSW-KRLS, where we take both the time value and correlation between samples into consideration.

## 4. Proposed SSW-KRLS Algorithm

Assume that there is a set of input–output pairs $D=\left\{u_{i},y_{i}\right\}{|}_{i=1}^{L}$ where *L* is the total number of samples. $u_{i}\in R^{m}$ are m-dimensional input vectors, and $y_{i}\in R$ is the output. In the traditional KRLS algorithm, in each time slot, when a new sample comes, the size dictionary increases, and this leads to an increase in computation and memory requirements. To solve this problem, we used a sliding-window approach to keep the size of the kernel dictionary within a fixed budget. Our criterion for discarding old samples was based on the correlation between existing samples in the kernel dictionary and the new sample. Moreover, we introduced a forgetting factor to exponentially weigh older data by scaling them, so as to track the dynamic characteristics of the channel.

In our proposed SSW-KRLS algorithm, we solved the following least-squares cost function:

(9) $$\min_{w}\sum_{j=1}^{i}\beta^{i-j}{\left(y_{j}-w^{T}\varphi_{j}\right)}^{2}+\lambda B\left(i\right)w^{T}w,$$

where β is the forgetting factor, and *i* is the iteration number, $y_{i}={\left(y_{1},...,y_{i}\right)}^{T}$ is the output vector, *w* is the weight vector, $B\left(i\right)=diag\left(\beta^{L-1},\beta^{L-2},\ldots ,1\right)$ is the forgetting matrix, $\varphi_{j}=\varphi \left(u_{j}\right)$ is the transformation of $u_{j}$, and λ is the regularization parameter. The optimal $w^{*}$ is solved:

(10) $$w^{*}={\left(\lambda B\left(i\right)+\Phi {}_{i}B\left(i\right)\Phi {}_{i}^{T}\right)}^{-1}\Phi {}_{i}B\left(i\right)y_{i}.$$

We reformulate the equation:

(11) $$w^{*}=\Phi {}_{i}{\left[\lambda B(i)+B\left(i\right)K_{i}\right]}^{-1}\bar{y_{i}},$$

where $\bar{y_{i}}=B\left(i\right)y_{i}$ is the exponentially weighted input signal.

**Figure 4:**
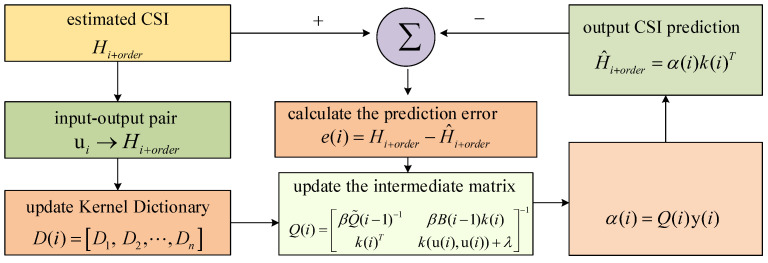
An illustration of the channel prediction algorithm steps.

The solutions for $Q\left(i\right)={\left[\lambda B(i)+B\left(i\right)K_{i}\right]}^{-1}$ are different under two cases in the *i*-th iteration: one case is that the size of the dictionary increased, and the other is that the size of the dictionary remained unchanged. Cases I and II are discussed in Section 4.2 and Section 4.3, respectively. In Section 4.1, we introduce how to update our dictionary when a new sample comes. Our methods are different depending on whether the size of the dictionary reaches the fixed sample budget, which is denoted by *M*. In particular, when the dictionary was not full, we discarded an old sample on the basis of the correlation analysis between the new and existing samples in the dictionary in order to slow down the increase in dictionary size. In the case when the size of the dictionary was full, we set a candidate discard set which contains the samples highly correlated with the new sample, and determine which sample to discard on the basis of their corresponding output. The whole process of our proposed algorithm is shown in Figure 4.

### 4.1. How to Optimally Discard an Old Sample

The cosine value is usually adopted for judging the correlation of two vectors. In the KRLS algorithm, on the other hand, the cosine value is calculated in the kernel Hilbert spaces. Suppose $u_{i}$ is a new sample, and $u_{j}$ is an existing sample in the dictionary. Let $k(u_{j})$ denote the kernel vector of $u_{j}\in D$. $k\left(u_{j}\right)={\left\{k_{jn}\right\}}_{n\neq i,j}$, where $k_{jn}=\kappa \left(u_{j},u_{n}\right)$. To measure the correlation between the existing and new samples, we calculated the cosine value of $k\left(u_{i}\right)$ and $k\left(u_{j}\right)$ for $\forall u_{j}\in D$, as $\cos\langle k\left(u_{i}\right),k\left(u_{j}\right)\rangle =\frac{k\left(u_{i}\right)k\left(u_{j}\right){}^{T}}{\left|k(u_{i})\right|\left|k(u_{j})\right|}$. When the size of the dictionary did not reach the budget, we found a sample with highest correlation with the new sample. Existing sample $u_{j}$ that had the highest cosine value was the most probable to be discarded, where $\underset{j}{j=\arg\max}\cos\langle k\left(u_{i}\right),k\left(u_{j}\right)\rangle$. We set a threshold τ and discarded sample $u_{j}$ if $\cos\langle k\left(u_{i}\right),k\left(u_{j}\right)\rangle \geq \tau$. The updated dictionary is:

(12) $$D\left(i\right)=\left\{\begin{array}{c} \left[D\left(i-1\right)\backslash u_{j}\;,\;\;u_{i}\right],if\max_{j}\cos\langle k\left(u_{i}\right),k\left(u_{j}\right)\rangle \geq \tau \\ \left[D\left(i-1\right)\;\;,u_{i}\right],\;\;\;if\max_{j}\cos\langle k\left(u_{i}\right),k\left(u_{j}\right)\rangle <\tau \end{array}\right.$$

In another case, when the size of the dictionary reaches the fixed budget, one sample must be discarded from the kernel dictionary in each time slot. In our strategy, we first set a candidate discard set on the basis of the correlation with the new sample, and then determined the optimal discarded sample according to its output value.

Candidate discard set *S* was composed of the samples that had high correlation with the new sample. Specifically, an existing sample in dictionary $u_{j}\in D$ was added into *S* if $\kappa (u_{i},u_{j})>\varepsilon$, where $u_{i}$ is a new sample and ε is a threshold. Among the samples in *S*, the one with the smallest value for prediction may be one that has the most similar information with the new sample or the one that is untypical with little probability to occur. We determined the discarded sample in combination with its corresponding output as following.

Suppose that the candidate discard set is $S=\left\{u_{1},u_{2},\ldots ,u_{n}\right\}$. The weighted average of the output values can be obtained as $y=\sum_{j=1}^{n}w_{j}y_{j}$ where

(13) $$w_{j}=\frac{\kappa (u_{i},u_{j})}{\sum_{j=1}^{n}\kappa (u_{i},u_{j})}$$

and *n* is the number of samples in *S*.

The deviation between the output value of $u_{j}\in S$ and the weighted average output value is $e_{j}=\left|y_{j}-y\right|$. The sample with maximal deviation $e_{\max}$ is not typical and may have occurred with a small probability, while the one with minimal deviation had similar information to that of the new sample. Suppose that the sample with the maximal deviation is $u_{j_{max}}$, and the sample with the minimal deviation is $u_{j_{min}}$. We chose one of these two samples to discard according to the production of $e_{\max}$ and $e_{\min}$. If the production of $e_{\max}$ and $e_{\min}$ was larger than threshold τ, this indicated that $e_{\max}$ was large enough, so we discarded $u_{j_{max}}$; otherwise, $e_{\min}$ was small enough, and we discarded $u_{j_{min}}$. The updated dictionary is:

(14) $$D\left(i\right)=\left\{\begin{array}{c} \left[D\left(i-1\right)\backslash u_{j_{max}}\;\;\;u_{i}\right],if\;e_{\max}e_{\min}\geq \tau \\ \left[D\left(i-1\right)\backslash u_{j_{min}}\;\;u_{i}\right],if\;e_{\max}e_{\min}<\tau . \end{array}\right.$$

### 4.2. Case I: The Size of D(i) Is Changed

As mentioned before, the prediction process depends on whether the size of the dictionary has increased or not. In Case I, when the size of dictionary increased, we could obtain from (11) that:

(15) $$\left\{\begin{array}{c} w_{i}=\Phi_{i}\alpha \left(i\right)\\ \alpha \left(i\right)=Q\left(i\right){\bar{y}}_{i}\\ Q\left(i\right)={\left[\lambda B\left(i\right)+B\left(i\right)K_{i}\right]}^{-1}. \end{array}\right.$$

With the combination of B(i) and $y_{i}$, we could obtain exponentially weighted input signal ${\bar{y}}_{i}$. We can find that

(16) $$B\left(i\right)=\left[\begin{array}{cc} \beta B(i-1) & 0\\ 0^{T} & 1 \end{array}\right]$$

(17) $$K_{i}=\left[\begin{array}{cc} K_{i-1} & h(i)\\ h{\left(i\right)}^{T} & \kappa (u_{i},u_{i}) \end{array}\right]$$

(18) $${\bar{y}}_{i}=\left[\begin{array}{c} \beta {\bar{y}}_{i-1}\\ {\bar{y}}_{i} \end{array}\right],$$

where $h\left(i\right)={\left[\kappa \left(u_{1},u_{i}\right),\kappa \left(u_{2},u_{i}\right),\ldots ,\kappa \left(u_{i-1},u_{i}\right)\right]}^{T}$. Thus

(19) $$B\left(i\right)K_{i}=\left[\begin{array}{cc} \beta B\left(i-1\right)K_{i-1} & \beta B(i-1)h(i)\\ h{\left(i\right)}^{T} & \kappa (u_{i},u_{i}) \end{array}\right]$$

By substituting (19) into (15), we can obtain that

(20) $$Q\left(i\right)={\left[\begin{array}{cc} \beta Q{\left(i-1\right)}^{-1} & \beta B(i-1)h(i)\\ h{\left(i\right)}^{T} & \kappa (u_{i},u_{i})+\lambda \end{array}\right]}^{-1}$$

With partitioned matrix inversion, we can obtain Q(i) in recursive form:

(21) $$Q\left(i\right)=r{\left(i\right)}^{-1}\left[\begin{array}{cc} \beta^{-1}r\left(i\right)Q\left(i-1\right)+\beta^{-1}z_{B}\left(i\right)z{\left(i\right)}^{T} & -z_{B}\left(i\right)\\ -\beta^{-1}z{\left(i\right)}^{T} & 1 \end{array}\right],$$

where

(22) $$\left\{\begin{array}{c} z_{B}\left(i\right)=Q\left(i-1\right)B\left(i-1\right)h\left(i\right)\\ r\left(i\right)=\kappa \left(u_{i},u_{i}\right)+\lambda -h{\left(i\right)}^{T}z_{B}\left(i\right)\\ z\left(i\right)=Q{\left(i-1\right)}^{T}h\left(i\right) \end{array}\right.$$

(23) $$\alpha \left(i\right)=\left[\begin{array}{c} \alpha \left(i-1\right)-z_{B}\left(i\right)r{\left(i\right)}^{-1}e\left(i\right)\\ r{\left(i\right)}^{-1}e\left(i\right) \end{array}\right],$$

and $e\left(i\right)=y_{i}-h{\left(i\right)}^{T}\alpha \left(i-1\right)$ is the prediction error in the ith time slot.

### 4.3. Case II: The Size of D(i) Is Unchanged

In the case when the size of the dictionary did not change, the information of the discarded sample $u_{j^{*}}$ in Q(i−1) needed to be deleted. The information of $u_{j^{*}}$ lay in the $j^{*}th$ column and $j^{*}th$ row of Q(i−1). In order to not influence the update of the matrix, we moved the $j^{*}th$ column and $j^{*}th$ row of Q(i−1) into the first row and the first column, and obtained $\hat{Q}\left(i-1\right)$. Correspondingly, we applied the same transformation to $K_{i-1}$ and obtained $\hat{K}\left(i-1\right)$. For $Q\left(i-1\right)={\left[\lambda B\left(i-1\right)+B\left(i-1\right)K_{i-1}\right]}^{-1}$; after the movement, the jth column that meets $j<j^{*}$ should be multiplied by β.

Suppose that the matrix after removing the first column and first row of $\hat{Q}\left(i-1\right)$ and $\hat{K}\left(i-1\right)$ is $\tilde{Q}\left(i-1\right)$ and $\tilde{K}\left(i-1\right)$, respectively. We can obtain its inversion $\tilde{Q}{\left(i-1\right)}^{-1}$ according to Appendix B,

(24) $$\hat{Q}\left(i-1\right)=\left[\begin{array}{cc} e & f^{T}\\ l & G \end{array}\right]$$

(25) $$\tilde{Q}{\left(i-1\right)}^{-1}=G-lf^{T}/e.$$

New matrix Q(i) can be formulated into a partitioned matrix:

(26) $$Q\left(i\right)={\left[\begin{array}{cc} \beta \tilde{Q}{\left(i-1\right)}^{-1} & \beta B\left(i-1\right)K_{i}\\ k{\left(i\right)}^{T} & \kappa (u_{i},u_{i})+\lambda \end{array}\right]}^{-1}={\left[\begin{array}{cc} A & b\\ p^{T} & \kappa (u_{i},u_{i})+\lambda \end{array}\right]}^{-1}.$$

Then, Q(i) can be obtained using the partitioned matrix:

(27) $$Q\left(i\right)={\left[\lambda \beta^{i}I\left(i\right)+B\left(i\right)K_{i}\right]}^{-1}=\left[\begin{array}{cc} A^{-1}\left(I+bp^{T}A^{-1}g\right) & -A^{-1}bg\\ -p^{T}A^{-1}g & g \end{array}\right],$$

where $A^{-1}=\beta^{-1}\tilde{K}{\left(i-1\right)}^{-1}B{\left(i-1\right)}^{-1}$, $g=(\kappa \left(u_{i},u_{i}\right)+\lambda -p^{T}A^{-1}{b)}^{-1}$.

Then, the weight coefficient is updated:

(28) $$\alpha \left(i\right)=Q\left(i\right){\bar{y}}_{i}=Q\left(i\right)B\left(i\right)y_{i},$$

where $y_{i}$ is composed of the output value of the samples $y_{i}={\left[y_{1},y_{2},\ldots ,y_{i}\right]}^{T}$. Lastly, we can obtain the prediction value for the next time slot as ${\hat{y}}_{i+1}=\alpha \left(i\right)k{\left(i\right)}^{T}$.

## 5. Performance Evaluation

Based on the analysis above, we present algorithmic steps as Algorithm Section 5 and in this section we show the simulation results of our proposed SSW-KRLS algorithmcompare its performance to that of the ALD-KRLS algorithm as a baseline. The basic simulation parameters are listed in Table 2. We adopted a 3D urban macro scenario, and considered a typical setting of 3 kHz with 30 kHz of subcarrier spacing. We considered 20 MHz of bandwidth that contained 51 resource partitions. Our adopted channel model was a CDL-A channel model, and the DL precoder was RZF.

**Algorithm 2** Proposed SSW-KRLS algorithm.
1:Initialize $Q\left(1\right)={\left(\lambda +\kappa (u_{1},u_{1})\right)}^{-1}$, $B\left(1\right)=\left[1\right]$, $\alpha \left(1\right)=Q\left(1\right)y_{1}$, $D\left(1\right)=\left[u_{1}\right]$.2:Step 1: Iterate for i>1: judging the number of samples in D(i), which is *L*. If L<M, perform Step 2; otherwise, perform Step 3.3:Step 2: For each sample $u_{j}$ in D(i−1), compute $\cos\langle k\left(u_{i}\right),k\left(u_{j}\right)\rangle$.Find $\underset{j}{j^{*}=\arg\max}\cos\langle k\left(u_{i}\right),k\left(u_{j}\right)\rangle$.If $\max_{j}\cos\langle k\left(u_{i}\right),k\left(u_{j}\right)\rangle >\tau$, discard $u_{j*}$. Then, add new sample $u_{i}$ into the dictionary. Turn to Step 4.4:Step 3: Construct candidate discard set *S*. Suppose $S=\left\{u_{1},u_{2},\ldots ,u_{n}\right\}$. Then, calculate the output of the samples.For each sample $u_{j}$, calculate $e_{j}=\left|y_{j}-y\right|$. Find $j_{max}=\underset{j}{\arg\max}e_{j}$ and $j_{min}=\underset{j}{\arg\min}e_{j}$.If $e_{\min}e_{\max}>\tau$, $D\left(i\right)=\left[D\left(i-1\right)\backslash u_{j_{max}},\;u_{i}\right]$.If $e_{\min}e_{\max}\leq \tau$, $D\left(i\right)=\left[D\left(i-1\right)\backslash u_{j_{min}},\;u_{i}\right]$.Turn to Step 4.5:Step 4: If D(i) is larger than D(i−1), perform Step 5; otherwise, perform Step 6.6:Step 5: Calculate Q(i) according to (21) and then calculate intermediate matrix $z_{B}\left(i\right)$, r(i), z(i) according to (22). Calculate α(i) according to (23). The prediction value for the next time slot is ${\hat{y}}_{i+1}=\alpha \left(i\right)k{\left(i\right)}^{T}$.7:Step 6: For Q(i−1) moving the $j^{*}th$ column and $j^{*}th$ row into the first column and the first row, and obtain $\tilde{Q}\left(i-1\right)$; calculate $\tilde{Q}{\left(i-1\right)}^{-1}$ by (24) and (25).Update Q(i) according to (26) and (27). Then, the prediction value is obtained on the basis of (28).


**Figure 5:**
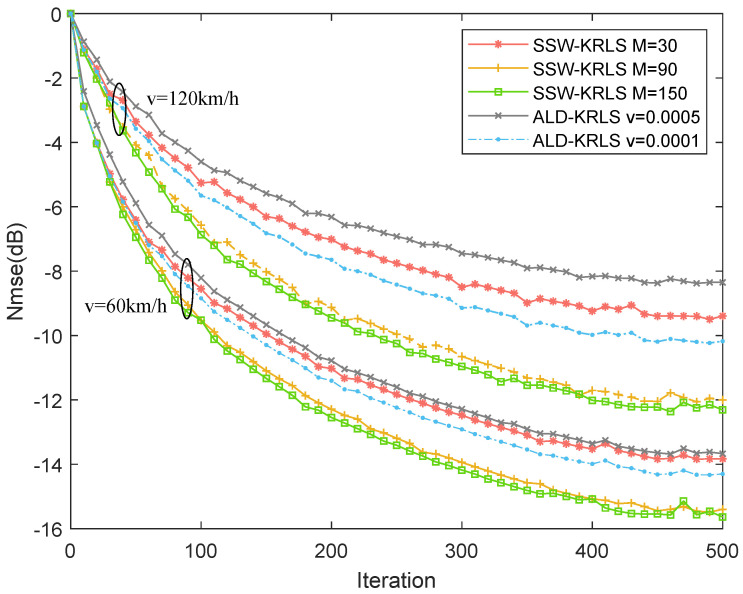
NMSE comparison of the proposed SSW-KRLS algorithm and the ALD-KRLS algorithm. β=0.97, $N_{t}=64$, $N_{r}=4$.

Figure 5 shows the normalized mean squared error (NMSE) performance of different algorithms. We show the performance of the ALD-KRLS and SSW-KRLS algorithms with the 60 and 120 km/h velocity levels for all UEs. When the UE speed was 60 km/h, the prediction algorithms was more accurate than with a UE speed of 120 km/h. Regarding the performance of the SSW-KRLS algorithm, we set different sample budgets: M=30,90,150. The algorithm performed better with a higher sample budget. The SSW-KRLS algorithm with sample budget M=30 performed better than the ALD-KRLS algorithm with v=0.001, and the SSW-KRLS algorithm with sample budget M=90 greatly outperformed the ALD-KRLS algorithm. However, the performance was insignificantly improved when changing the sample budget from 90 to 150.

**Figure 6:**
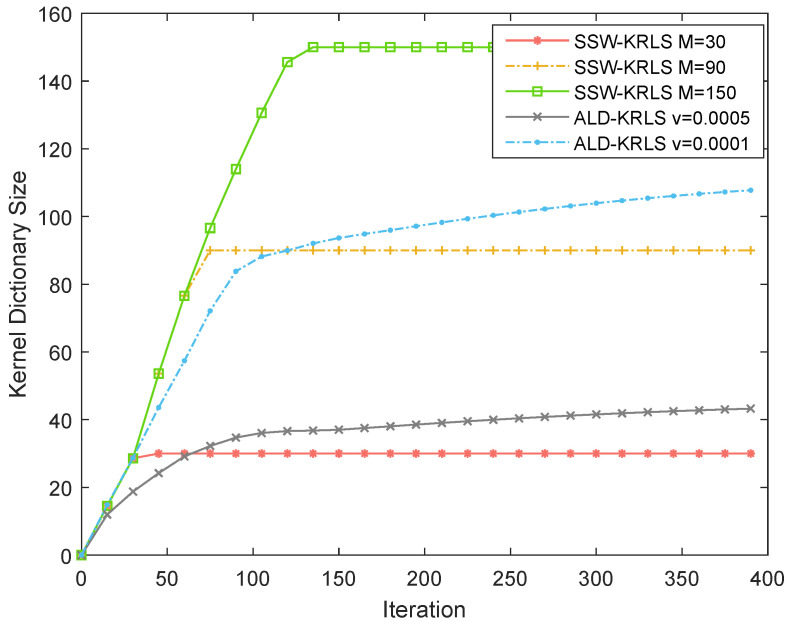
The kernel dictionary size of the proposed SSW-KRLS algorithm and ALD-KRLS algorithm varies with the number of iterations. β=0.97, $N_{t}=64$, $N_{r}=4$.

Figure 6 shows the kernel dictionary size with 400 iterations. The kernel dictionary size for all algorithms first grew and then slowed down; the size for SSW-KRLS remained unchanged. The SSW-KRLS algorithm with sample budget M=90 outperformed the ALD-KRLS algorithm with v=0.001, while the former used fewer samples. This indicates the superiority of our proposed SSW-KRLS algorithm.

**Figure 7:**
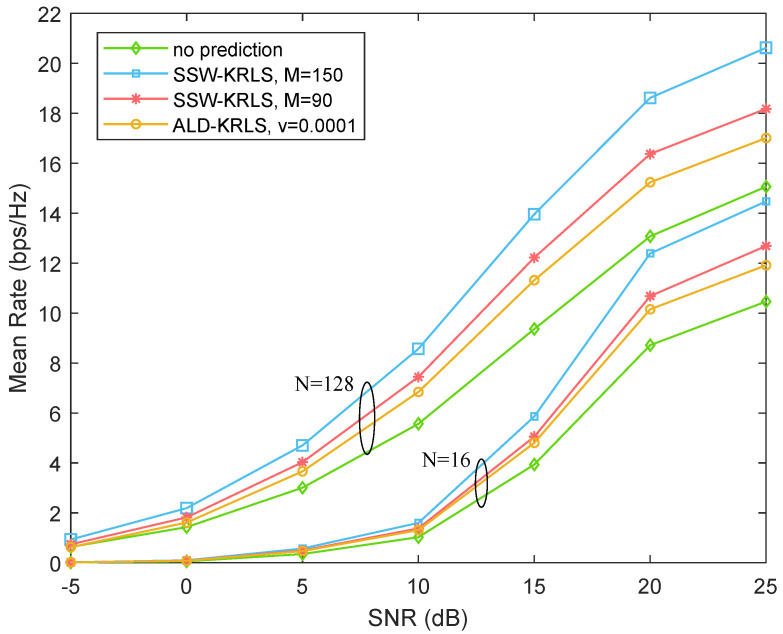
Performance comparison among no prediction (last uplink measurement), the ALD-KRLS algorithm, and the proposed SSW-KRLS algorithm. β=0.97, v=60 km/h.

**Figure 8:**
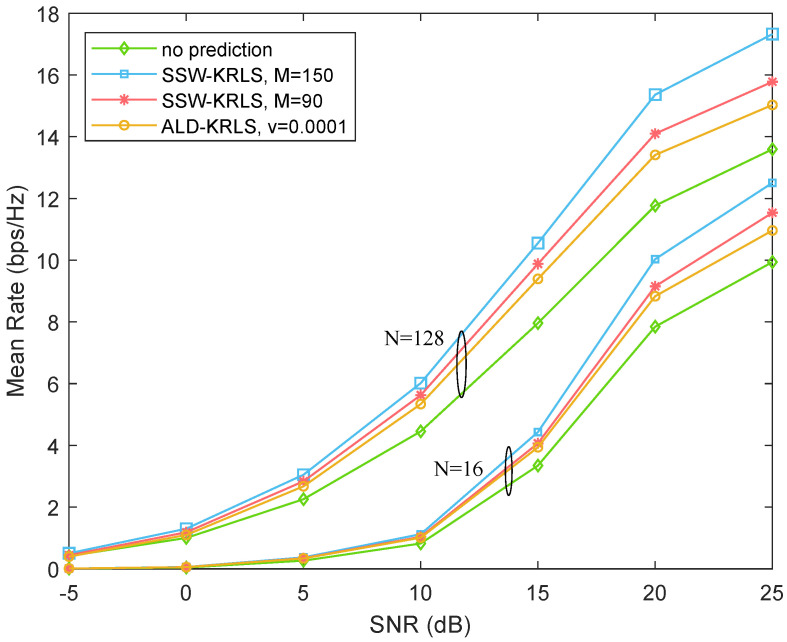
Performance comparison among no prediction (last uplink measurement), the ALD-KRLS algorithm, and the proposed SSW-KRLS algorithm. β=0.97, v=120 km/h.

Figure 7 and Figure 8 show the mean rate of mobile users under different algorithms at the speed of 60 and 120 km/h, respectively. Comparing the figure at two speed levels shows that the user with the lower speed had a higher mean rate. In addition, the performance could be enhanced greatly with the use of the channel prediction algorithm. Particularly, our proposed SSW-KRLS algorithm with sample budgets M=150 and M=90 achieved better performance than that of the ALD-KRLS algorithm with v=0.0001; for our proposed SSW-KRLS algorithm, a higher sample budget brought about better performance. Moreover, users with more antennas showed better performance under any circumstances.

## 6. Conclusions

This paper proposed a new sparse sliding-window KRLS algorithm where a candidate discard set is selected through correlation analysis between the mapping vectors in kernel Hilbert spaces of the new input sample and the existing samples in the kernel dictionary. It then determines the discarded sample in combination with its corresponding output to achieve dynamic sample updates. Specifically, the proposed SSW-KRLS algorithm, which maintains the size of the kernel dictionary within the sample budget, requires a fixed amount of memory and computation per time step, incorporates regularization, and achieves online predictions. Moreover, in order to sufficiently track strongly changeable dynamic characteristics, a forgetting factor was considered in the proposed algorithm. Numerical simulations demonstrated that, under a realistic channel model of 3GPP in a rich scattering environment, our proposed algorithm achieved superior performance in terms of both predictive accuracy and kernel dictionary size than that of the ALD-KRLS algorithm. The NMSE for the channel prediction of the SSW-KRLS algorithm with M=90 was about 2 dB lower than that of the ALD-KRLS algorithm with v=0.001, while the kernel dictionary was 17% smaller.

## Figures and Tables

**Table 1:** Some common sparseness criteria.

Criterion	Indicators	Handling Method
ALD	Determine whether the kernel function of the new sample can be linearly represented by the kernel function of the existing samples in the dictionary: $\delta \left(n\right)=\min_{a}{∥\sum_{i=1}^{m}a\left(i\right)\varphi \left(x_{i}\right)-\varphi \left(x_{n}\right)∥}^{2}$	If δ(n)<τ, discard new samples. If δ(n)≥τ, add the new sample to the dictionary.
NC	Calculate the minimal distance between the new and existing samples in the kernel dictionary: $dis=\min_{a}{∥\sum_{i=1}^{m}a\left(i\right)\varphi \left(x_{i}\right)-\varphi \left(x_{n}\right)∥}^{2}$	If dis<τ, discard new samples. If dis≥τ, add the new sample to the dictionary.
SC	According to information theory, based on prior joint Gaussian distribution, the amount of information brought by the new sample is: $S\left(n\right)=-\ln\;\;p\left(x_{n},d_{n}\left|D_{n-1}\right.\right)$, where $p(x_{n},d_{n}\left|D_{n-1}\right.)$ is posterior probability distribution of $\left(x_{n},d_{n}\right)$	If $\tau_{1}<S\left(n\right)<\tau_{2}$, add the new sample to the dictionary. If $S\left(n\right)>\tau_{2}$, discard new samples.
CC	Calculate the maximal kernel function of the new and existing samples : $\mu =\max_{i}\left|k(x_{i},x_{n})\right|$	If μ>τ, discard new samples. If μ<τ, add the new sample to the dictionary.

**Table 2:** Some common sparseness criteria.

Scenario	3D Urban Macro (3D UMa)
Carrier frequency	3 kHz
Subcarrier spacing	30 kHz
Bandwidth	20 MHz
Channel model	CDL-A
Delay spread	100 ns
DL precoder	RZF
order	5

## Appendix A

For a non-singular matrix *A*, if there is a new column and a new row added to the matrix and get *E* as as followings:

(A1) $$E=\left[\begin{array}{cc} A & b\\ b^{T} & c \end{array}\right].$$

Suppose the inversion of *E* is formulated as:

(A2) $$E^{-1}=\left[\begin{array}{cc} D & e\\ e^{T} & f \end{array}\right].$$

Then the $E^{-1}$ can be calculated by solving:

(A3) $$\left\{\begin{array}{c} AD+be^{T}=I\\ Ae+bf=0\\ b^{T}e+cf=1 \end{array}\right.,$$

where $E^{-1}$ can be obtained as:

(A4) $$E^{-1}=\left[\begin{array}{cc} A^{-1}\left(I+bb^{T}A^{-1H}f\right) & -A^{-1}bf\\ -{\left(A^{-1}b\right)}^{T}f & f \end{array}\right],$$

where $f={\left(c-b^{T}A^{-1}b\right)}^{-1}$.

## Appendix B

For a non-singular matrix *E*, if the first column and the first row are removed from the matrix and get *C*:

(A5) $$E=\left[\begin{array}{cc} a & b^{T}\\ b & C \end{array}\right].$$

Suppose the inversion of *E* is formulated as:

(A6) $$E^{-1}=\left[\begin{array}{cc} d & e^{T}\\ e & F \end{array}\right].$$

Then the $C^{-1}$ can be calculated by solving:

(A7) $$\left\{\begin{array}{c} bd+Ce=0\\ be^{T}+CF=I\prime \end{array}\right.$$

where $C^{-1}$ can be obtained as $C^{-1}=F-ee^{T}/d$.
